# Crystal structure of barium perchlorate anhydrate, Ba(ClO_4_)_2_, from laboratory X-ray powder data

**DOI:** 10.1107/S2056989015008828

**Published:** 2015-05-09

**Authors:** Jeonghoo H. Lee, Ji Hoon Kang, Sung-Chul Lim, Seung-Tae Hong

**Affiliations:** a Daegu Gyeongbuk Institute of Science & Technology (DGIST), Daegu 711-873, Republic of Korea

**Keywords:** crystal structure, powder diffraction, Ba(ClO_4_)_2_, barium perchlorate anhydrate

## Abstract

The previously unknown crystal structure of barium perchlorate anhydrate, determined and refined from laboratory X-ray powder diffraction data, represents a new structure type. The title compound was obtained by heating hydrated barium perchlorate [Ba(ClO_4_)_2_·*x*H_2_O] at 423 K *in vacuo* for 6 h. It crystallizes in the ortho­rhom­bic space group *Fddd*. The asymmetric unit contains one Ba (site symmetry 222 on special position 8*a*), one Cl (site symmetry 2 on special position 16*f*) and two O sites (on general positions 32*h*). The structure can be described as a three-dimensional polyhedral network resulting from the corner- and edge-sharing of BaO_12_ polyhedra and ClO_4_ tetra­hedra. Each BaO_12_ polyhedron shares corners with eight ClO_4_ tetra­hedra, and edges with two ClO_4_ tetra­hedra. Each ClO_4_ tetra­hedron shares corners with four BaO_12_ polyhedra, and an edge with the other BaO_12_ polyhedron.

## Abstract 2

The previously unknown crystal structure of barium perchlorate anhydrate, determined and refined from laboratory X-ray powder diffraction data, represents a new structure type. The structure can be described as a three-dimensional polyhedral network resulting from the corner- and edge-sharing of BaO_12_ polyhedra and ClO_4_ tetra­hedra.

## Chemical context   

**Figure 1:**
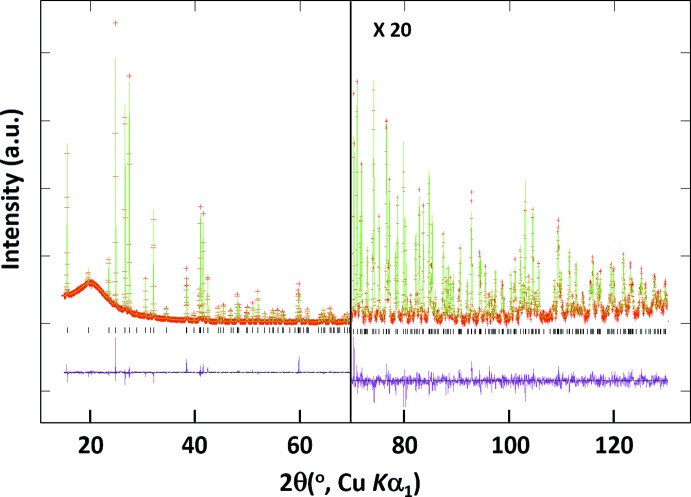
X-ray Rietveld refinement profiles for Ba(ClO_4_)_2_ recorded at room temperature. Crosses mark experimental points (red) and the solid line is the calculated profile (green). The bottom trace shows the difference curve (purple) and the ticks denote expected peak positions.

The alkaline earth metal ions (Mg, Ca, Sr and Ba) have been of increasing inter­est as ion carriers for post Li ion batteries (Wang *et al.*, 2013 ▸), and their perchlorates are often used as conventional organic electrolyte salts for electrochemical cells such as magnesium (Amatucci *et al.*, 2001 ▸; Levi *et al.*, 2010 ▸) and calcium ion batteries (Padigi *et al.*, 2015 ▸). Since such salts adsorb water easily from the atmosphere and the water causes unwanted side reactions in the electrochemical cells, removing water from the salts and its confirmation before use would be very important. However, due to the difficulty in growing a single crystal of such anhydrous perchlorates, no crystal structure had ever been solved before we first identified the magnesium perchlorate structure from powder X-ray diffraction data (Lim *et al.*, 2011 ▸). Barium perchlorate is a very strong oxidizing agent due to the high oxidation state of chlorine VII, and it is commonly stabilized as hydrate forms in the atmos­phere. Several different forms of the hydrates are expected to exist, as observed in the magnesium analogues (Robertson & Bish, 2010 ▸; West, 1935 ▸). The crystal structure of the trihydrate form was determined from single-crystal data (Gallucci & Gerkin, 1988 ▸), but the anhydrous form, Ba(ClO_4_)_2_, has not been reported to date. We present here its crystal structure, as determined and refined from laboratory powder X-ray diffraction data (Fig. 1 ▸). This is the second crystal structure reported among the anhydrate alkaline earth metal perchlor­ates.

## Structural commentary   

**Figure 2:**
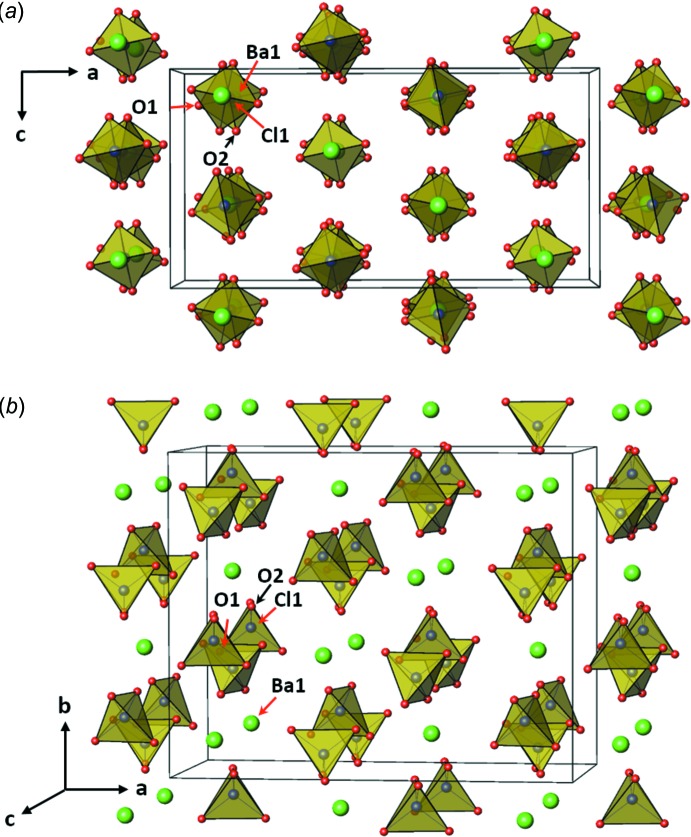
The unit cell structures for Ba(ClO_4_)_2_ with (ClO_4_) tetra­hedra (yellow) and Ba atoms (green), showing (*a*) the [010] view and (*b*) the [001] view.

**Figure 3:**
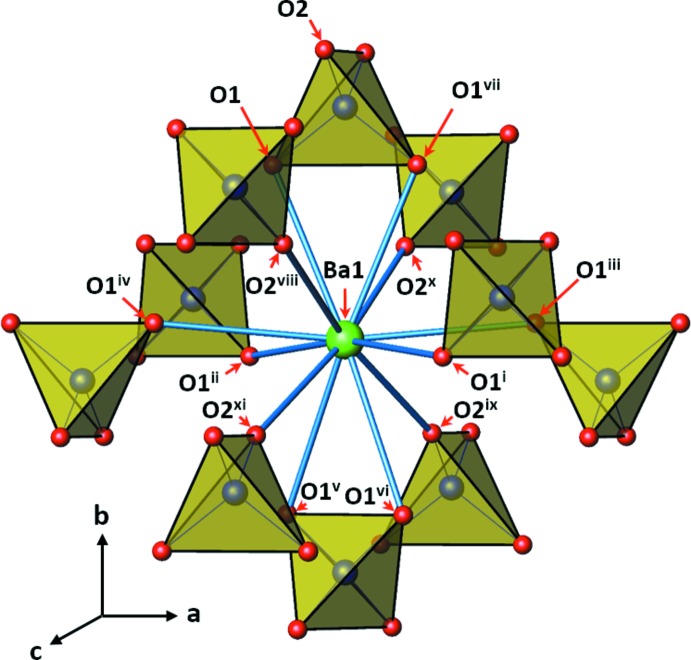
The local environment of the Ba^2+^ cation (green sphere) surrounded by (ClO_4)_ tetra­hedra (yellow). [Symmetry codes: (i) *x* + 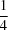, *y* − 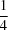, −*z* + 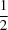; (ii) −*x*, *y* − 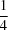, *z* − 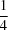; (iii) *x* + 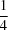, −*y* + 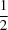, *z* − 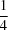; (iv) −*x*, −*y* + 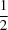, −*z* + 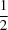; (v) *x*, −*y* + 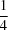, −*z* + 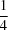; (vi) −*x* + 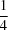, −*y* + 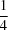, *z*; (vii) −*x* + 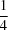, *y*, −*z* + 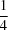; (viii) *x*, −*y* + 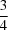, −*z* + 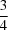; (ix) *x*, *y* − 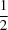, *z* − 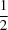; (x) −*x* + 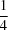, −*y* + 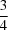, *z* − 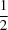; (xi) −*x* + 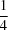, *y* − 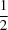, −*z* + 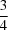.]

Anhydrous Ba(ClO_4_)_2_ crystallizes in a new structure type in terms of atomic ratios (1:2:8) and its polyhedral network is, to our knowledge, unique. The asymmetric unit contains one Ba (site symmetry 222 on special position *8a*), one Cl (site symmetry 2 on special position 16*f*) and two O sites (on general positions 32*h*). The crystal structure is illustrated in Fig. 2 ▸, where two different views along [010] and [001] are presented for better visualization. The crystal structure is represented with ClO_4_ tetra­hedra and Ba atoms in Fig. 2 ▸ *a* and 2*b*. The local environment around the Ba atom is presented in Fig. 3 ▸. It is clearly seen that there are chains of [(ClO_4_)–Ba–(ClO_4_)]_∞_ parallel to the *b*-axis direction. Along each chain, the barium atom is placed between the two ClO_4_ tetra­hedra, bonded to two oxygen atoms at each tetra­hedron. The [010] view in Fig. 2 ▸ *a* clearly shows the two-dimensional arrangement of the chains. The chains are inter­connected through Ba—O bonds. Each chain is surrounded by six neighboring ones that are shifted parallel to *b*-axis in such a way that a barium atom of the central chain is connected to the oxygen atoms of eight ClO_4_ tetra­hedra of six neighboring chains. Four tetra­hedra are from four chains, one from each. The other four tetra­hedra are from two other chains, two from each. The structure may also be described as a three-dimensional polyhedral network resulting from the corner- and edge-sharing of BaO_12_ polyhedra and ClO_4_ tetra­hedra. Each BaO_12_ polyhedron shares corners with eight ClO_4_ tetra­hedra, and edges with two ClO_4_ tetra­hedra. Each ClO_4_ tetra­hedron shares corners with four BaO_12_ polyhedra, and an edge with the other BaO_12_ polyhedron. The oxygen atoms in a ClO_4_ tetra­hedron consist of two O1 and two O2 ones. O1 is bonded to three atoms, one Cl and two Ba atoms, forming an almost planar environment. On the other hand, O2 is bonded to only two atoms, Cl and Ba. Selected bond lengths are given in Table 1 ▸.

It is inter­esting to see the significant difference in crystal structures between Ba(ClO_4_)_2_ and Mg(ClO_4_)_2_ due to the difference in the cation radii, 1.61 Å for Ba^2+^ and 0.72 Å for Mg^2+^ (Shannon, 1976 ▸). The much bigger cation, Ba^2+^, is coordinated by eight ClO_4_ tetra­hedra, while the magnesium is coordinated by only six. Accordingly, the repulsion between two cations of Ba^2+^–Cl^7+^ must be much weaker that that of the magnesium compound since the inter­atomic Ba—Cl distances of 3.55–4.06 Å are much longer than that (3.3 Å) of Mg—Cl for the same charges. This might be a reason why magnesium perchlorate is much more highly reactive with water when exposed to the atmosphere.

The empirical expression for bond valence, which has been widely adopted to estimate valences in inorganic solids (Brown, 2002 ▸), was used to check the Ba(ClO_4_)_2_ crystal structure. The bond-valence sums (Brown & Altermatt, 1985 ▸; Brese & O’Keeffe, 1991 ▸) calculated with the program *Valence* (Hormillosa *et al.*, 1993 ▸) [given in v.u. (valence units): Ba 2.20, Cl 6.89, O1 2.04 and O2 1.73] match the expected charges of the ions reasonably well.

## Synthesis and crystallization   

The anhydrous form of barium perchlorate was prepared by dehydration from Ba(ClO_4_)_2_·xH_2_O (97%, Aldrich). The powder was thoroughly ground in an agate mortar and put into the bottom of a fused-silica tube with the other end sealed with a rubber septum. The tube was inserted into a box furnace through a hole on top of the furnace so that the bottom of the tube was at the center of the furnace inside, and the other end outside connected to a vacuum pump through a needle stuck into the septum. It was heated at a rate of 4K/min up to 423K for 6 h under continuous vacuum. After furnace cooling, powder sampling for X-ray measurement was processed in an Ar atmosphere glove-box, and a tightly sealed dome-type X-ray sample holder commercially available from Bruker was used to prevent hydration during measurement.

## Refinement details   

Crystal data, data collection and structure refinement details are summarized in Table 2 ▸. The powder X-ray diffraction (XRD) data were collected at room temperature on a Bragg–Brentano diffractometer (PANalytical Empyrean) with a Cu *K*α1 X-ray tube, a focusing primary Ge (111) monochromator (λ = 1.54059 Å), and a position-sensitive PIXcel 3D 2x2 detector, the angular range of 15 ≤ 2θ ≤ 130°, step 0.0260 and total measurement time of 13 h at room temperature. The structure determination from the powder XRD data was performed using a combination of the powder profile refinement program *GSAS* (Larson & Von Dreele, 2000 ▸) and the single-crystal structure refinement program *CRYSTALS* (Betteridge *et al.*, 2003 ▸). For a three-dimensional view of the Fourier density maps, *MCE* was used (Rohlíček & Hušák, 2007 ▸). The XRD pattern was indexed using the program *TREOR90* (Werner, 1990 ▸) run in *CRYSFIRE* (Shirley, 2002 ▸) *via* the positions of 20 diffraction peaks, resulting in an ortho­rhom­bic unit cell. The systematic absences suggested the space group *Fddd*. The structure determination was performed in the same way as in our previous work (Lee & Hong, 2008 ▸) where the details were described. At the beginning, a structural model with only a dummy atom at an arbitrary position in the unit cell was used. Structure factors were extracted from the powder data, then direct methods were used for the initial solution of the structure using *SHELXS97* (Sheldrick, 2008 ▸) run in *CRYSTALS*, which yielded a couple of atom positions. However, not all the atoms could be identified at once. The partial model at this stage replaced the initial dummy-atom model, and was used for a Le Bail fit in *GSAS*. Then, improved structure factors were extracted, which were used for the improved data in the refinement in *CRYSTALS*. These processes were iterated until a complete and satisfactory structural model was obtained. Finally, Rietveld refinement was employed to complete the structure determination, resulting with reasonable temperature factors and an *R_wp_* factor of 0.06.

## Supplementary Material

Crystal structure: contains datablock(s) I. DOI: 10.1107/S2056989015008828/cv5487sup1.cif

Rietveld powder data: contains datablock(s) I. DOI: 10.1107/S2056989015008828/cv5487Isup2.rtv

Structure factors: contains datablock(s) I. DOI: 10.1107/S2056989015008828/cv5487Isup3.hkl

CCDC reference: 1063587

Additional supporting information:  crystallographic information; 3D view; checkCIF report

## Figures and Tables

**Table 1:** Selected bond lengths ()

Ba1O1	2.901(4)	Ba1O2^iii^	2.903(4)
Ba1O1^i^	2.939(4)	Cl1O1	1.441(4)
Ba1O1^ii^	2.901(4)	Cl1O2	1.437(4)

Symmetry codes: (i) 
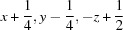
; (ii) 
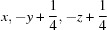
; (iii) 
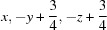
.

**Table 2:** Experimental details

Crystal data	
Chemical formula	Ba(ClO_4_)_2_
*M*_r_	336.23
Crystal system, space group	Orthorhombic, *F* *d* *d* *d*
Temperature (K)	298
*a*, *b*, *c* ()	14.304(9), 11.688(7), 7.2857(4)
*V* (^3^)	1218.1(11)
*Z*	8
Radiation type	Cu *K*_1_, = 1.54059
Specimen shape, size (mm)	Flat sheet, 20 20

Data collection	
Diffractometer	PANalytical Empyrean
Specimen mounting	Packed powder
Data collection mode	Reflection
Scan method	Step
2 values ()	2_min_ = 14.992 2_max_ = 129.964 2_step_ = 0.026

Refinement	
*R* factors and goodness of fit	*R*_p_ = 0.041, *R*_wp_ = 0.060, *R*_exp_ = 0.045, *R*(*F* ^2^) = 0.05733, ^2^ = 1.769
No. of data points	4423
No. of parameters	25

Computer programs: *X’Pert Data Collector* and *X’Pert HighScore-Plus* (PANalytical, 2011 ▸), *GSAS* (Larson Von Dreele, 2000 ▸), *SHELXS97* (Sheldrick, 2008 ▸), *CRYSTALS* (Betteridge *et al.*, 2003 ▸) and *ATOMS* (Dowty, 2000 ▸).

## supplementary crystallographic information

### Crystal data

**Table:**

Ba(ClO_4_)_2_	*Z* = 8
*M**_r_* = 336.23	*F*(000) = 1232.0
Orthorhombic, *F**d**d**d*	*D*_x_ = 3.667 Mg m^−^^3^
Hall symbol: -F 2uv 2vw	Cu *K*α_1_ radiation, λ = 1.54059 Å
*a* = 14.304 (9) Å	*T* = 298 K
*b* = 11.688 (7) Å	white
*c* = 7.2857 (4) Å	flat sheet, 20 × 20 mm
*V* = 1218.1 (11) Å^3^	

### Data collection

**Table:**

PANalytical Empyrean diffractometer	Data collection mode: reflection
Radiation source: sealed X-ray tube, PANalytical Cu Ceramic X-ray tube	Scan method: step
Specimen mounting: packed powder	2θ_min_ = 14.992°, 2θ_max_ = 129.964°, 2θ_step_ = 0.026°

### Refinement

**Table:**

Least-squares matrix: full	Profile function: CW Profile function number 3 with 19 terms Pseudovoigt profile coefficients as parameterized in P. Thompson, D.E. Cox & J.B. Hastings (1987). J. Appl. Cryst.,20,79-83. Asymmetry correction of L.W. Finger, D.E. Cox & A. P. Jephcoat (1994). J. Appl. Cryst.,27,892-900. #1(GU) = 0.000 #2(GV) = 0.000 #3(GW) = 0.000 #4(GP) = 4.256 #5(LX) = 1.263 #6(LY) = 7.277 #7(S/L) = 0.0005 #8(H/L) = 0.0005 #9(trns) = -1.26 #10(shft)= 1.5725 #11(stec)= 4.60 #12(ptec)= 1.24 #13(sfec)= 0.00 #14(L11) = -0.018 #15(L22) = -0.022 #16(L33) = -0.202 #17(L12) = 0.017 #18(L13) = 0.017 #19(L23) = 0.000 Peak tails are ignored where the intensity is below 0.0010 times the peak Aniso. broadening axis 0.0 0.0 1.0
*R*_p_ = 0.041	25 parameters
*R*_wp_ = 0.060	0 restraints
*R*_exp_ = 0.045	(Δ/σ)_max_ = 0.01
*R*(*F*^2^) = 0.05733	Background function: GSAS Background function number 1 with 36 terms. Shifted Chebyshev function of 1st kind 1: 567.648 2: -857.136 3: 662.653 4: -377.264 5: 131.512 6: 59.5938 7: -169.995 8: 206.701 9: -185.404 10: 133.783 11: -68.7256 12: 6.71856 13: 43.4515 14: -72.2732 15: 82.7653 16: -73.3661 17: 50.0183 18: -22.8495 19: -2.57480 20: 20.6662 21: -29.1651 22: 28.9267 23: -24.2542 24: 14.4066 25: -5.32227 26: -4.03875 27: 10.7050 28: -13.4416 29: 11.1646 30: -9.08855 31: 2.53787 32: -0.292410 33: -1.46976 34: 0.544854 35: -1.31862 36: 0.893355
χ^2^ = 1.769	Preferred orientation correction: March-Dollase AXIS 1 Ratio= 0.97385 h= 1.000 k= 0.000 l= 0.000 Prefered orientation correction range: Min= 0.96103, Max= 1.08275
4423 data points	

### Fractional atomic coordinates and isotropic or equivalent isotropic displacement parameters (Å^2^)

**Table:**

	*x*	*y*	*z*	*U*_iso_*/*U*_eq_	
Ba1	0.125	0.125	0.125	0.0139 (2)*	
Cl1	0.125	0.42875 (18)	0.125	0.0160 (7)*	
O1	0.0471 (3)	0.3533 (3)	0.1575 (5)	0.0162 (11)*	
O2	0.1412 (3)	0.5016 (4)	0.2807 (4)	0.0170 (12)*	

### Geometric parameters (Å, º)

**Table:**

Ba1—O1	2.901 (4)	Ba1—O2^viii^	2.903 (4)
Ba1—O1^i^	2.939 (4)	Ba1—O2^ix^	2.903 (4)
Ba1—O1^ii^	2.939 (4)	Ba1—O2^x^	2.903 (4)
Ba1—O1^iii^	2.939 (4)	Ba1—O2^xi^	2.903 (4)
Ba1—O1^iv^	2.939 (4)	Cl1—O1	1.441 (4)
Ba1—O1^v^	2.901 (4)	Cl1—O1^vii^	1.441 (4)
Ba1—O1^vi^	2.901 (4)	Cl1—O2	1.437 (4)
Ba1—O1^vii^	2.901 (4)	Cl1—O2^vii^	1.437 (4)
			
O1—Ba1—O1^i^	110.93 (10)	O1^i^—Ba1—O2^ix^	110.87 (10)
O1—Ba1—O1^ii^	78.56 (7)	O1^i^—Ba1—O2^x^	125.15 (10)
O1—Ba1—O1^iii^	106.64 (7)	O1^i^—Ba1—O2^xi^	63.76 (10)
O1—Ba1—O1^iv^	63.56 (12)	O1^vi^—Ba1—O1^vii^	134.82 (15)
O1—Ba1—O1^v^	134.82 (15)	O2^xii^—Ba1—O2^ix^	170.85 (15)
O1—Ba1—O1^vi^	170.64 (14)	O2^viii^—Ba1—O2^x^	120.42 (15)
O1—Ba1—O1^vii^	46.25 (15)	O2^viii^—Ba1—O2^xi^	60.43 (15)
O1—Ba1—O2^viii^	60.21 (10)	O1—Cl1—O1^vii^	104.5 (3)
O1—Ba1—O2^ix^	123.92 (10)	O1—Cl1—O2	110.96 (19)
O1—Ba1—O2^x^	65.35 (10)	O1—Cl1—O2^vii^	111.6 (2)
O1—Ba1—O2^xi^	110.84 (10)	O2—Cl1—O2^vii^	107.3 (3)
O1^i^—Ba1—O1^ii^	170.09 (14)	Ba1—O1—Ba1^xiii^	116.44 (12)
O1^i^—Ba1—O1^iii^	66.21 (14)	Ba1—O1—Cl1	104.6 (2)
O1^i^—Ba1—O1^iv^	114.73 (14)	Ba1^xiii^—O1—Cl1	133.1 (2)
O1^i^—Ba1—O2^viii^	60.66 (10)	Ba1^viii^—O2—Cl1	164.5 (2)

Symmetry codes: (i) *x*+1/4, *y*−1/4, −*z*+1/2; (ii) −*x*, *y*−1/4, *z*−1/4; (iii) *x*+1/4, −*y*+1/2, *z*−1/4; (iv) −*x*, −*y*+1/2, −*z*+1/2; (v) *x*, −*y*+1/4, −*z*+1/4; (vi) −*x*+1/4, −*y*+1/4, *z*; (vii) −*x*+1/4, *y*, −*z*+1/4; (viii) *x*, −*y*+3/4, −*z*+3/4; (ix) *x*, *y*−1/2, *z*−1/2; (x) −*x*+1/4, −*y*+3/4, *z*−1/2; (xi) −*x*+1/4, *y*−1/2, −*z*+3/4; (xii) *x*, −*y*+7/4, −*z*+11/4; (xiii) −*x*, *y*+1/4, *z*+1/4.
